# High-Resolution Imaging of Parafoveal Cones in Different Stages of Diabetic Retinopathy Using Adaptive Optics Fundus Camera

**DOI:** 10.1371/journal.pone.0152788

**Published:** 2016-04-08

**Authors:** Mohamed Kamel Soliman, Mohammad Ali Sadiq, Aniruddha Agarwal, Salman Sarwar, Muhammad Hassan, Mostafa Hanout, Frank Graf, Robin High, Diana V. Do, Quan Dong Nguyen, Yasir J. Sepah

**Affiliations:** 1 Ocular Imaging Reading and Research Center, Stanley M. Truhlsen Eye Institute, University of Nebraska Medical Center, Omaha, NE, United States of America; 2 Department of Ophthalmology, Assiut University, Assiut, Egypt; 3 College of Public Health, University of Nebraska Medical Center, Omaha, NE, United States of America; Justus-Liebig-University Giessen, GERMANY

## Abstract

**Purpose:**

To assess cone density as a marker of early signs of retinopathy in patients with type II diabetes mellitus.

**Methods:**

An adaptive optics (AO) retinal camera (rtx1^**™**^; Imagine Eyes, Orsay, France) was used to acquire images of parafoveal cones from patients with type II diabetes mellitus with or without retinopathy and from healthy controls with no known systemic or ocular disease. Cone mosaic was captured at 0° and 2°eccentricities along the horizontal and vertical meridians. The density of the parafoveal cones was calculated within 100×100-μm squares located at 500-μm from the foveal center along the orthogonal meridians. Manual corrections of the automated counting were then performed by 2 masked graders. Cone density measurements were evaluated with ANOVA that consisted of one between-subjects factor, stage of retinopathy and the within-subject factors. The ANOVA model included a complex covariance structure to account for correlations between the levels of the within-subject factors.

**Results:**

Ten healthy participants (20 eyes) and 25 patients (29 eyes) with type II diabetes mellitus were recruited in the study. The mean (± standard deviation [SD]) age of the healthy participants (Control group), patients with diabetes without retinopathy (No DR group), and patients with diabetic retinopathy (DR group) was 55 ± 8, 53 ± 8, and 52 ± 9 years, respectively. The cone density was significantly lower in the moderate nonproliferative diabetic retinopathy (NPDR) and severe NPDR/proliferative DR groups compared to the Control, No DR, and mild NPDR groups (*P* < 0.05). No correlation was found between cone density and the level of hemoglobin A_1c_ (HbA_1c_) or the duration of diabetes.

**Conclusions:**

The extent of photoreceptor loss on AO imaging may correlate positively with severity of DR in patients with type II diabetes mellitus. Photoreceptor loss may be more pronounced among patients with advanced stages of DR due to higher risk of macular edema and its sequelae.

## Introduction

The underlying pathophysiological processes that result in visual loss in diabetic retinopathy (DR) are yet to be completely understood.[1] Although development of microvascular complications contributes significantly to vision loss, evidence suggests that DR has a neurodegenerative component that may also contribute to vison loss as well. [2, 3] Neurodegenerative changes include apoptosis of several populations of retinal cells, including photoreceptors, bipolar cells, ganglion cells, and astrocytes.[4–7]There is evidence to suggest that structural and functional impairments of these cell lines may not only precede microangiopathy but also contribute to the earliest alterations of the vascular structures.[8] For instance, early signs of neuronal dysfunction such as loss of contrast and color sensitivity may be seen 2 years after the diagnosis of diabetes [9, 10], whereas, it may take up to 10–15 years for the microvascular changes to develop.[11] Similarly, Du et al. have demonstrated that early microangiopathic changes in diabetic eyes may be due to the oxidative stress and inflammation of photoreceptors associated with diabetes.[12]

While there is plenty of evidence to suggest that changes in the photoreceptors layer may be present in patients with diabetes who have not yet shown clinical signs of retinopathy, it has been challenging to study such changes in vivo until recently. Advances in retinal imaging techniques and development of more sophisticated optical systems that incorporate the principles of adaptive optics (AO) can allow a clinician to capture enface images of photoreceptors in near histological resolution. Imaging systems based on AO principles correct for aberration arising from various refractive surfaces within the eye. Such correction consequently leads to a high-resolution imaging that allows noninvasive in vivo visualization of the retinal cells, which has been only feasible in histological studies until recently.[13] So far, AO has been used to study the state of various cellular and vascular structures of the retina, in particular photoreceptors, in both health and disease.[14–16]The index study aims to assess cone density as a marker of early signs of retinopathy in patients with type II diabetes mellitus.

## Materials and Methods

The study was conducted in accordance with the Declaration of Helsinki and was approved by the institutional review board (IRB) of the University of Nebraska Medical Center. A written informed consent was obtained from all study participants after explaining the imaging procedure and study aim.

### Study Participants

Healthy volunteers with no known ocular or systemic diseases and patients with diagnosis of type II diabetes mellitus who received retina and optometry services at the Stanley M. Truhlsen Eye Institute were included in the study. Eligibility for study participation was confirmed by comprehensive ocular examination. Study participants were divided into three groups: i.e. Control (healthy volunteers), No DR (patients with diabetes with no retinopathy), and DR (patients with diabetic retinopathy). In turn, the DR group was divided into the following subgroups: mild nonproliferative diabetic retinopathy (NPDR), moderate NPDR, severe NPDR, or proliferative diabetic retinopathy (PDR); the latter 2 groups were combined into severe NPDR/PDR group for statistical analysis. Eyes with high myopia (>10 diopters), media opacity, pseudophakia, active macular edema, hemorrhage, exudate or scar in the central 2 mm of the fovea, and any concurrent retinal disease other than DR were excluded from the study. To confirm the absence of macular edema in patients with moderate NPDR, severe NPDR, and PDR, Spectral Domain Optical Coherence Tomography (SD-OCT) was performed. Macular edema in patients with no signs of maculopathy in the central 2 mm in the No DR and mild NPDR groups and was ruled out using clinical examination with slit-lamp biomicroscopy only. All eyes in the severe NPDR and PDR groups and 2 eyes in the moderate NPDR group had a previous history of macular edema that had been treated with intravitreal anti-vascular endothelial growth factor (VEGF) prior to recruitment in the study. Severity of DR was classified based on Early Treatment of Diabetic Retinopathy Severity Scale.

### Image Acquisition and Analysis

High-resolution retinal images were acquired using the rtx1^**™**^ adaptive optics retinal flood-illumination camera (Imagine Eyes, Orsay, France). The imaging device is a noncontact en face imaging system that is composed of 3 main components: high resolution fundus camera, Shack–Hartmann wave-front sensor, and a deformable mirror, which permits real time correction of the aberration of the outgoing ocular wavefront. The camera uses an infrared illumination (wavelength of 850 nm) and has a resolution of approximately 2-μm. The field of view is 4×4° that corresponds approximately to 1.2×1.2 mm square on the retinal surface based on the axial length of the eye. Axial length measurements of all study participants were performed using a noncontact biometry (IOL Master^®^; Carl Zeiss Meditech, Germany).

Images from eyes with dilated pupils were acquired using the following standardized protocol. Study participants were instructed to fixate on an internal yellow fixation cross. A video camera incorporated in rtx1 device, which give a real-time display of eye movement, was used to monitor fixation of the patients during image acquisition. A set of 40 images was acquired over 4 seconds. The degree of AO correction shown on the camera software panel was confirmed to be <1 mrad before image acquisition to ensure a reasonable correction of the optical aberration and better acquisition quality. Cone mosaic was imaged at 0° and 2° eccentricity along the horizontal and vertical meridians. The foveal reference point of each patient was located by finding the central point of the image taken while the patient was fixating at an internal fixation cross with coordinates set at 0° angle (x = 0° and y = 0°). Eccentricity along the orthogonal meridians was measured as the distance between the area of interest and the foveal reference point.

**Fig 1:**
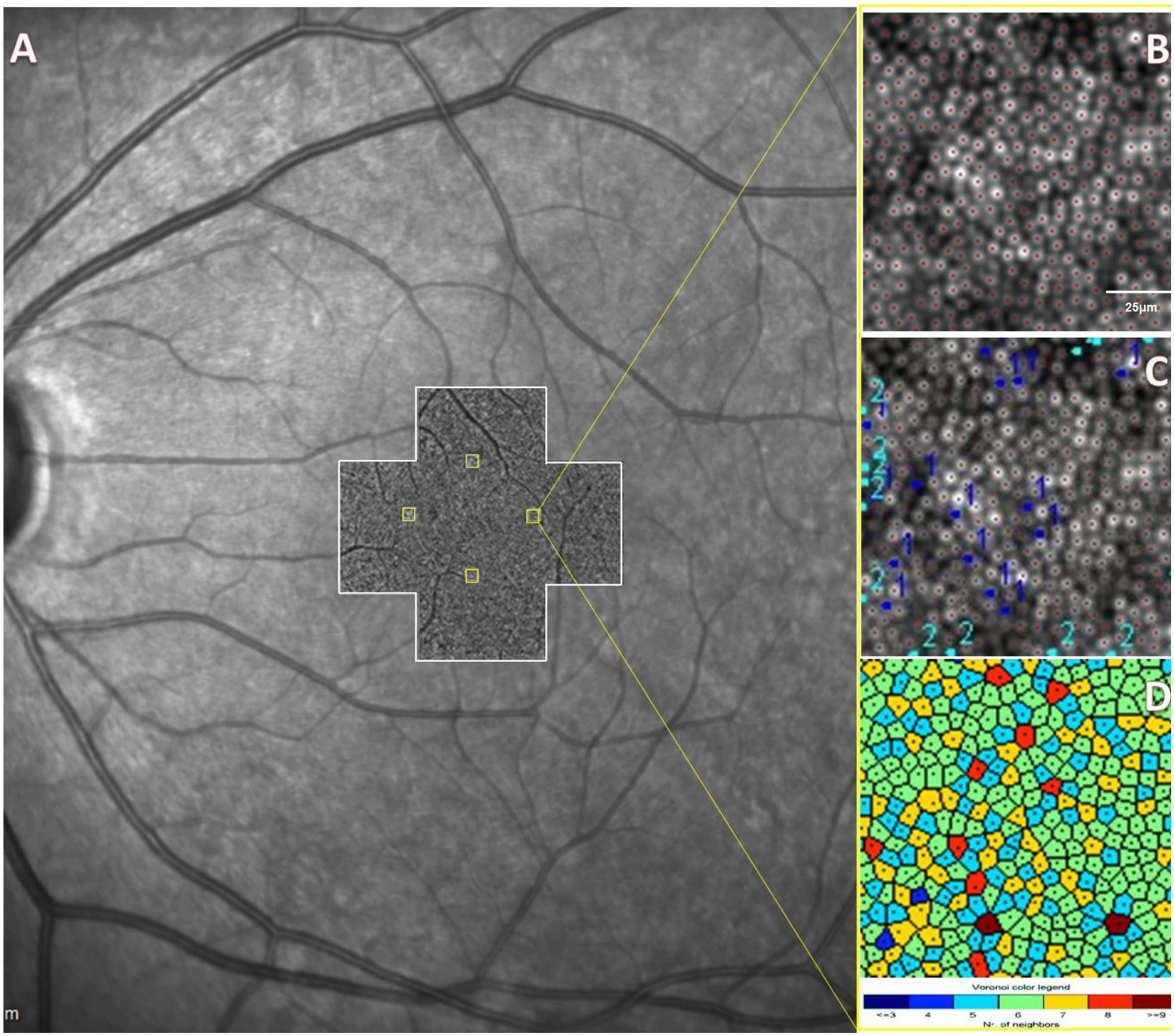
Position of the Regions of Interest (ROIs) with Respect to the Fovea. A: Scanning laser ophthalmoscope (SLO) image of the left eye of a healthy control. AO images (white-bordered cross centered at the fovea), taken at 0° and 2° along the vertical and horizontal meridians, are stitched and superimposed on the SLO image. Four 100×100-μm squares are placed 500-μm (yellow squares) from the foveal center within the 4 retinal quadrants. B: Magnified AO image of the temporal 500-μm eccentricity square. AOdetect^®^ software recognizes and counts the cells marked by red dots. C: Example of manual correction of the automated counts by one grader (1, missed cones; 2, cones erroneously counted by the automated software). D: Color map of Voronoi tiles.

Analysis of the cone mosaic was performed using proprietary software provided by the manufacturer (AOdetect v0.1, Imagine Eyes, Orsay, France) at the Ocular Imaging Reading and Research Center. Initially, acquired images were processed by the software to produce a 4×4° high-contrast image of the retina with an improved signal-to-noise ratio and a resolution of 0.8 μm/pixel. Then, the same software was used to analyze cone density, cone spacing, and Voronoi domains of the selected regions of interest (ROIs) after correction for the axial length of the eye.[17] The ROI could be moved within 50 μm if necessary to avoid blood vessels. A 100×100- μm sampling window size was chosen, which approximately corresponds to the area of retina stimulated by Goldmann size III target. The ROIs were positioned so that the distance between the center of the sampling window and the foveal reference point is 500-μm (yellow squares) across all 4 retinal quadrants, i.e., 4 regions per eye were selected (Fig 1). Cone density values were manually corrected by 2 masked graders (MK and MH). ImageJ (V1.48, National Institute of Health, USA), an open source software, was used to perform manual corrections. First, the ROI was magnified by the zoom function to facilitate identification of individual cones. Second, the cell counter plugin was used to manually add missed cones and/or subtract erroneously counted ones by the automated software. Cones at the edges of the image that are not completely visible were excluded. The steps involved in manual correction using ImageJ are described in S1 Appendix. Cone spacing, measured from the center of one photoreceptor to the center of the closest photoreceptor, was calculated automatically.

### Statistical Analysis

Descriptive analyses of all variables that were visually assessed for outliers were conducted. Four measurements of cone density, the primary response variable, were collected from each individual. These measurements were defined by within-subject factors, the location of an ROI (lower, upper, nasal, temporal). These measurements (i.e., dependent data) were evaluated with a repeated measures analysis of variance (ANOVA) that consisted of one between-subjects factor, stage of retinopathy (Control, No DR, Mild NPDR, Moderate NPDR, and Severe NPDR/PDR) and the within-subject factors.

In addition, the measurements were collected from both eyes in a subset of the subjects: 4 subjects in the diabetic groups with both eyes at the same level of retinopathy and 10 control subjects with fellow eyes. Since multiple measurements were collected from each participant, the repeated measures analysis of variance (ANOVA) model included a complex covariance structure to account for correlations between the levels of the within-subject factors. The 3 design factors were evaluated as fixed effects in the repeated measures ANOVA, and *P* values for main effects and interactions were evaluated. *P* values for differences in means were adjusted due to multiple comparisons. In addition, a repeated measures analysis of covariance model (ANCOVA) evaluated the relationship between cone density and duration of diabetes or control of diabetes (measured as the level of hemoglobin A_1c_ [HbA_1c_]) for participants with diabetes. Statistical analyses were generated with SAS/STAT software, Version 9.4 (2002–2012 SAS Institute Inc.).

## Results

### Patient Characteristics

Ten healthy participants (20 eyes) and 25 patients (29 eyes) with type II diabetes mellitus were recruited in the study. The age and axial length were not significantly different between the study groups. Among those with diabetes, 7 patients (9 eyes) did not have retinopathy, 6 patients (7 eyes) had mild NPDR, 7 patients (8 eyes) had moderate NPDR, 3 patients (3 eyes) had severe NPDR and 2 patients (2 eyes) had PDR. The mean (± standard deviation [SD]) age of the Control, No DR, and DR groups was 55 ± 8, 53 ± 8, and 52 ± 9 years, respectively. Best corrected visual acuity (BCVA) was 20/20 among controls and ranged from 20/20–20/70 among patients with diabetes. The mean (± SD) HbA_1c_ was 9.4 ± 2.7% and 9.1 ± 2.3% in the No DR and DR groups, respectively. The baseline characteristics of the study participants are shown in Table 1.

**Table 1:** Baseline Characteristics of Study Participants.

	Control	No DR	Mild NPDR	Moderate NPDR	Severe NPDR—PDR
Eyes, number	20	9	7	8	5
Age, mean ± SD, years	55 ± 8	53 ± 8	50 ± 9	52 ± 8	55 ± 10
Female gender, %	80	67	50	43	40
Axial length, mean ± SD, mm	23.4 ±1	23.2 ±1	23.9 ±1	23.7 ±1	23.8 ±1
Duration of Diabetes, mean ± SD, years	N/A	10 ± 3	15 ± 10	18 ± 8	16 ± 6

SD: standard deviation

### Cone Density

Cone density was not significantly different between the 4 retinal quadrants (Table 2); therefore, the cone density values at the four quadrants of each retinal eccentricity were averaged (Table 3). The mean (± SD) cone density in the Control and No DR groups was 27642 ± 3043 and 25932 ± 2532 cells/mm^2^. In the DR groups, the mean cone density (± SD) ranged between 25164 ± 2344 and 20528 ± 1791 cells/mm^2^.

**Table 2:** Cone Density in Each Quadrant Across the Study Groups.

Quadrants	Controls	No DR	Mild NPDR	Moderate NPDR	Severe NPDR—PDR
Inferior	27829 ±3181	26154 ±2271	25204 ±2319	21531 ±2564	19700 ±2057
Nasal	27587 ±2716	25694 ±2532	25130 ±2701	21825 ±2873	19430 ±1552
Temporal	27979 ±3248	26034 ±2807	25512 ±1997	22219 ±3168	21523 ±2292
Superior	27274 ±2996	25848 ±2490	24810 ±2305	21731 ±2230	21460 ±971

All values are presented as mean ± standard deviation. Cone density unit is cone/mm^2^.

**Table 3:** Average Cone Density, Spacing, and Voronoi (Mean ± SD) at 500-μm Eccentricity.

Study Groups	Average Cone Density ^a^, cone/mm^2^	Spacing, μm	Voronoi, 6 tiles
Controls	27642 ±3043	7.0 ± 0.6	44.8% ± 1.7
No DR	25932 ±2532	7.1 ± 0.5	45.6% ± 0.8
Mild NPDR	25164 ±2344	7.3 ± 0.5	43.4% ± 2.9
Moderate NPDR	21827 ±2731	8.5 ± 0.7	40.3% ± 3.4
Severe NPDR—PDR	20528 ±1791	8.5 ± 0.7	40.0% ± 3.9

**NPDR**: Non proliferative diabetic retinopathy; **PDR**: Proliferative diabetic retinopathy.

^a^ Average cone density is the mean of cone density values in the 4 quadrants.

The cone density was significantly lower in the combined diabetic group (No DR and DR groups) compared to Control group (*P* < 0.05) at the 500-μm retinal eccentricity (Table 4). A significant decline (P<0.05) in the cone density was observed in the moderate NPDR and severe NPDR/PDR groups compared to the Control, No DR, and mild NPDR groups (Table 4). No statistically significant difference was observed between the Control, No DR, and mild NPDR groups.

**Table 4:** Cone Density Comparisons at 500-μm Eccentricity.

	Stage of retinopathy	Mean^a^, cone/mm^2^	Difference	Standard Error	*P* Value
Control group	Control / Diabetic Retinopathy groups	2.94/2.50	0.44	0.09	<0.001*
	Control/ No DR	2.78/2.58	0.205	0.122	0.45
	Control/Mild NPDR	2.78/2.53	0.247	0.128	0.31
	Control/Moderate NPDR	2.78/2.19	0.591	0.122	<0.001*
	Control/Severe NPDR-PDR	2.78/2.07	0.708	0.138	<0.001*
No DR group	No DR/Mild NPDR	2.58/2.53	0.042	0.137	1.00
	No DR/Moderate NPDR	2.58/2.19	0.386	0.132	0.038*
	No DR/Severe NPDR-PDR	2.58/2.07	0.503	0.147	<0.010*
Mild NPDR group	Mild NPDR/ Moderate NPDR	2.53/2.19	0.344	0.137	0.100
	Mild NPDR/ Severe NPDR-PDR	2.53/2.07	0.461	0.152	0.028*
Moderate NPDR group	Moderate NPDR/ Severe NPDR-PDR	2.19/2.07	0.117	0.147	0.93

^a^ Values are presented in thousands.

* Statistically significant difference between the groups (*P* < 0.05).

**NPDR**: Non proliferative diabetic retinopathy; **PDR**: Proliferative diabetic retinopathy.

**Fig 2:**
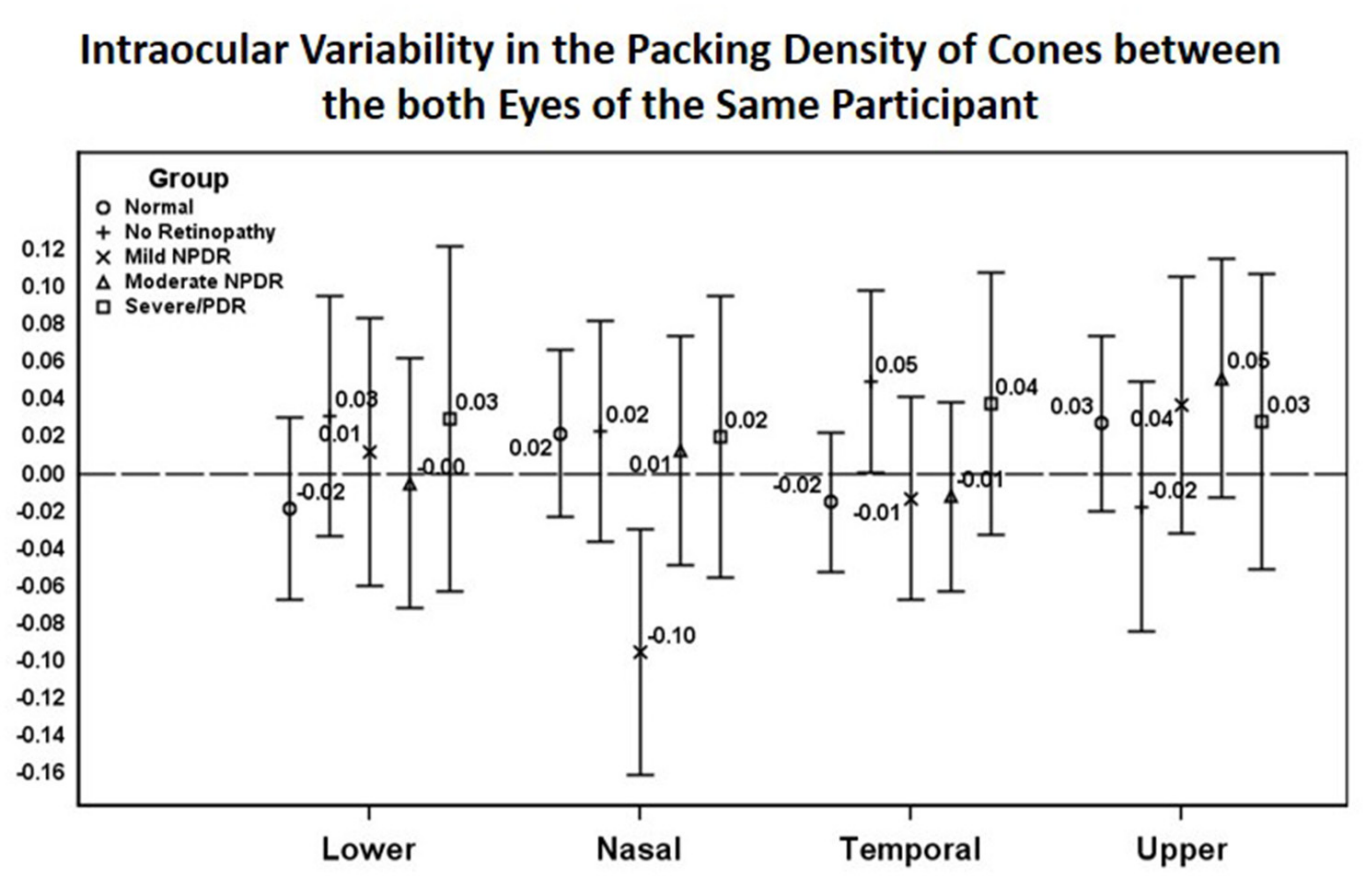
Intraocular Variability in the Packing Density of Cones between the both Eyes of the Same Participant.

The variability of cone density between both eyes of the same participant was not significantly different across the 4 quadrants in any of the study groups (in the DR group, both eyes were at the same severity of retinopathy). This finding had 2 exceptions: the temporal 500-μm eccentricity in the No DR group and the nasal 500-μm eccentricity in the mild NPDR group showed significant variability between the two eyes as shown in Fig 2.

There was no correlation between cone density at different stages of retinopathy and the level of HbA_1c_ or the duration of diabetes. The inter-observer agreement among all groups was 0.79.

### Inter-photoreceptor Distance

The mean (± SD) inter-photoreceptor distance in the Control and No DR groups was 7.0 ± 6 and 7.1 ± 0.5 μm, respectively. In the DR groups, the mean (± SD) inter-photoreceptor distance ranged between 7.3± 0.5 and 8.5 ± 0.7 μm (Table 3).

The inter-photoreceptor distance did not show statistically significant difference across the vertical and horizontal meridians in the Control (*P* > 0.05), No DR (*P* > 0.05), and DR groups (P > 0.05). Statistically significant difference in the inter-photoreceptor spacing between different study groups are represented in Table 5.

**Table 5:** Cone Spacing Comparisons at 500-μm Eccentricity*.

	Stage of retinopathy	Mean, μm	Difference	Standard Error	*P* Value
Control group	Control/Moderate NPDR	7.07/8.31	- 1.243	0.304	<0.003
	Control/Severe NPDR-PDR	7.07/8.48	- 1.416	0.346	<0.003
No DR group	No DR/Moderate NPDR	7.16/8.31	- 1.150	0.330	0.012
	No DR/Severe NPDR-PDR	7.16/8.48	- 1.322	0.369	<0.010

* Presented data are restricted to the pairs of groups with significantly different means (*P* < 0.05).

**NPDR**: Non proliferative diabetic retinopathy; **PDR**: Proliferative diabetic retinopathy.

### Voronoi Quantification

Cone packing regularity was assessed through analysis of Voronoi domains. The mean percentage of cones with hexagonal Voronoi tiles in the Control and No DR groups was 44.8% and 45.6%, respectively. In the DR groups, the percentage of cones with hexagonal Voronoi tiles ranged between 43.4% and 40.0%.

With inclusion of cones having 5 and 7 Voronoi tiles, the mean percentage increased to 92.7%, 93.4%, and 90.0% in the Control, No DR, and DR groups, respectively.

Mean Voronoi values (6 tiles) of the study groups were compared: healthy controls and patients without DR tended to have higher mean Voronoi values than did patients with DR, especially those at the more advanced DR stages (Table 6).

**Table 6:** Voronoi (6 tiles) Comparisons at 500-μm Eccentricity*.

	Stage of retinopathy	Mean	Difference	Standard Error	*P* Value
Control group	Control/Moderate NPDR	44.9/39.8	5.08	1.66	0.038
	Control/Severe NPDR-PDR	44.9/38.9	6.00	1.85	0.024
No DR group	No DR/Moderate NPDR	45.6/39.8	5.83	1.84	0.029
	No DR/Severe NPDR-PDR	45.6/38.9	6.76	2.01	0.018

* Data are restricted to the pairs of groups with significantly different means (*P* < 0.05).

**NPDR**: Non proliferative diabetic retinopathy; **PDR**: Proliferative diabetic retinopathy.

**Fig 3:**
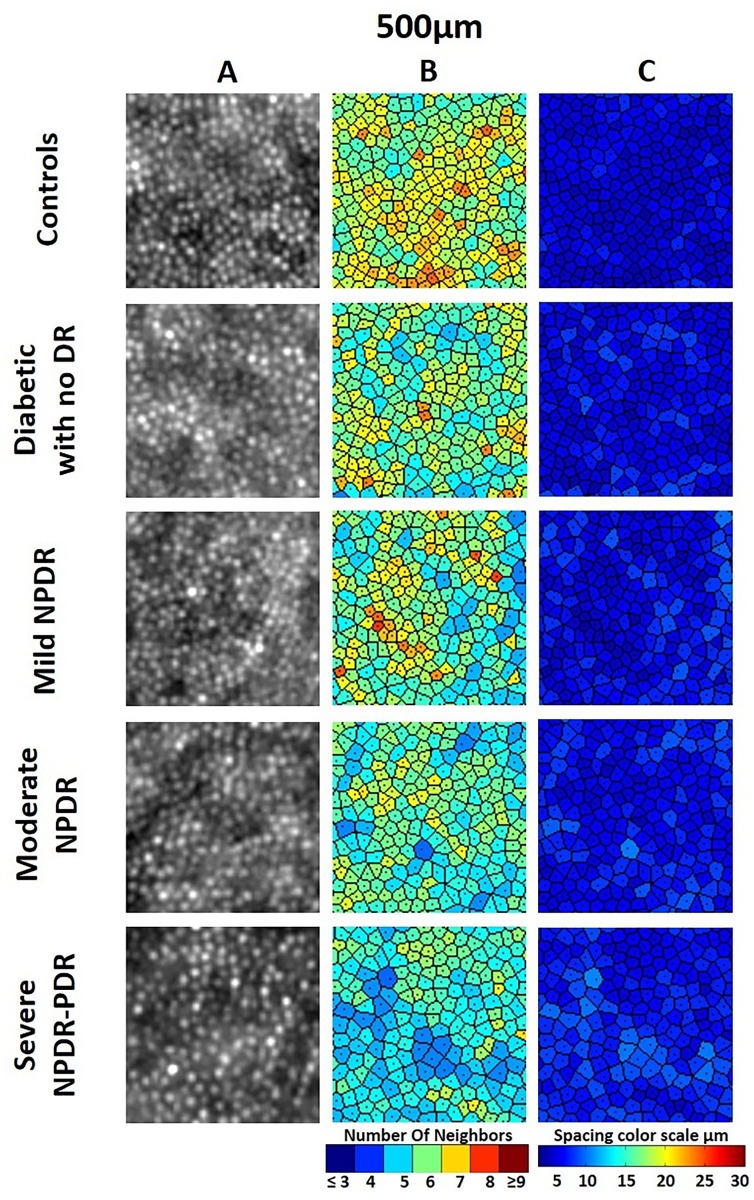
Images of Parafoveal Cone Mosaic at 500-μm Eccentricity Representative of Each Study Group. A: Adaptive optics image of the cone mosaic in 100-μm 100-μm sampling window. B: Corresponding color map of Voronoi tiles. C: Corresponding cone map.

Representative images of cone density, cone spacing, and Voronoi across study groups are shown in Fig 3.

## Discussion

We used AO fundus camera to assess and compare cone density in age-matched healthy volunteers and patients with type II diabetes mellitus. Our findings indicate that parafoveal cone density decreased by a mean (± SD) of 1672 ± 2859 cones/mm^2^ per step of DR progression. This decrease in parafoveal cone density was not associated with changes in the duration of diabetes or serum HbA_1c_ levels. Similar to our findings, a previous AO study revealed a decline in the photoreceptor counts in patients with type I diabetes mellitus at early stages of DR (No DR and mild NPDR) vs healthy controls [18]. However, in contrast to our findings, photoreceptor decline reported in that study was significant and was associated with changes in serum HbA_1c_ levels. Another AO study evaluated the photoreceptors density at slightly more peripheral locations (~7° from the fovea) in adolescents and young adults with type I diabetes and no DR. [19] This study reported no significant difference in cone density vs controls. However, this study was limited to patients with no DR with a relatively younger age of diagnosis of diabetes.

Decrease in cone density was most prominent in the moderate NPDR and combined severe NPDR/PDR groups. The loss of photoreceptors in patients with advanced DR may be due to history of DME. DME is known to cause thinning and disruption of the retinal layers, particularly the photoreceptor layer.[20] Hence, DME may have contributed to photoreceptor loss in these patients. In addition, decreased cone density may be linked to the prior use of anti-VEGF therapy in these patients. Since VEGF is known to have a neuroprotective effect on photoreceptors, anti-VEGF agents may potentially have a deleterious effect on photoreceptors. [21, 22] However, we have also observed cone loss at the earlier stages of DR, in the absence of either significant involvement of retinal vasculature or prior anti-VEGF use. This observation suggests that other factors may also contribute to photoreceptor damage.[23] Individual cone densities in the right vs left eye were not statistically different across study groups, with minor exceptions.

Cone photoreceptor counts in the Control group reported in the present study fall within the range reported in previous studies. Several discrepancies in cone counts exist between previous histological and AO studies of photoreceptor topography. For instance, in the comprehensive histological study, Curcio et al report the density of cones of approximately 34,000 cones/mm^2^ at 0.5 mm eccentricity from the fovea.[24] However, previous experiments on postmortem eyes have reported significant variation in the foveal cone density.[24–27] In AO studies, cone density at different retinal quadrants has been reported to range from approximately 31,000 to 23,000 cones/mm^2^ at approximately 2 degree from the foveal center.[28–36] Variability of cone density between different studies remained even after correction for axial length and other confounding factors.[37–39]. This variability could be attributed to several factors, including the lack of a standardized approach to cone counting and differences in image processing software, AO systems, sampling window size, and foveal reference point location. High intersubject variability of cone density may also play a significant role.[24, 40] While discrepancy exists between our findings and some earlier studies, data from other reports support our findings. For example, data from 192 eyes of healthy volunteers revealed an average cone density of approximately 30,000 cones/mm2 at 500-μm eccentricity from the fovea, which falls within a reasonable agreement with values we obtained from the cohort of healthy individuals at 500-μm eccentricity from the fovea with higher mean age of our study population.[34]

We compared cone densities across the 4 meridians and observed no significant difference. Park et al have obtained similar results across 2 eccentricities, at 500um and 1500um from the foveal center.[34] This contrasts with finding of two histological studies, which showed asymmetry of cone density in different quadrants; however, whether the asymmetry is statistically significant remains unclear from the studies.[24, 37] Song et al have shown statistically significant difference across the 4 meridians in their AO study; however, cone density was analyzed at all eccentricities combined as opposed to each eccentricity.[39] Further studies are needed to describe cone densities across retinal meridians.

We have identified several possible limitations of this study: resolution of AO system, study size, and the presence of several sources of confounding or bias. Firstly, the resolution of rtx1 camera, which was used in present study, is insufficient to assess the density of extremely tightly packed cones at the center of the fovea. Whether or not this loss of parafoveal cones reflects similar changes at the foveal center remains unclear. To date, the association of photoreceptor loss and vision loss in patients with DR remains obscure. Secondly, small sample size is another limitation of this prospective observational cohort study. Recruitment of patients to this study was challenging because patients with type II diabetes mellitus usually present with some degree of lens opacification or pseudophakia that interferes with AO image acquisition and quality. In addition, the presence of intraretinal edema adds to the challenge by interfering with AO imaging in these patients. Lastly, imbalanced gender distribution across the study groups and history of DME in certain patients, which may be associated with loss of photoreceptors and defective AO imaging as its sequelae, are possible sources of bias. A clinical study conducted by Park et al reported no significant difference in cone density between genders; however, based on the limited data available, the possibility of gender bias in this study remains.[34] Possible confounding factors are the prior use of anti-VEGF therapies and history of macular edema, which may have a pathological effect on photoreceptors, in some of the study participants.

In conclusion, the extent of photoreceptor loss may correlate positively with severity of DR in patients with type II diabetes mellitus. We did not find a significant difference in the cone densities between the Control and No DR groups; however, we did observe a trend towards lower cone density in the DR group. Patients with advanced stages of DR may suffer loss of photoreceptors due to higher risk of macular edema and its sequelae. Detection of photoreceptor loss at early stages of DR and better understanding of the impact of photoreceptor loss on the microvascular changes and visual function may contribute to changing the current standard regimen of treatment via earlier intervention to stop further damage. We hope that future studies conducted by us or other investigators will assess the progression of photoreceptor loss over time. This may provide more insight into the magnitude of photoreceptor loss at different stages of DR, which is an important measure of DR pathology and a potential therapeutic target.

## Supporting Information

S1 Appendix Steps for manual correction using ImageJ. (PDF)
