# Supplementary material for: High-Resolution Imaging of Parafoveal Cones in Different Stages of Diabetic Retinopathy Using Adaptive Optics Fundus Camera
Source: PLoS One. 2016 Apr 8;11(4):e0152788. doi: 10.1371/journal.pone.0152788 (PMC4825992; doi:10.1371/journal.pone.0152788)

Appendix S1. Steps for manual correction using ImageJ

1) Load the ROI and magnify it.

Firstly open ImageJ and load the ROI image, then use the zoom icon in ImageJ to magnify the image to facilitate cone identification..

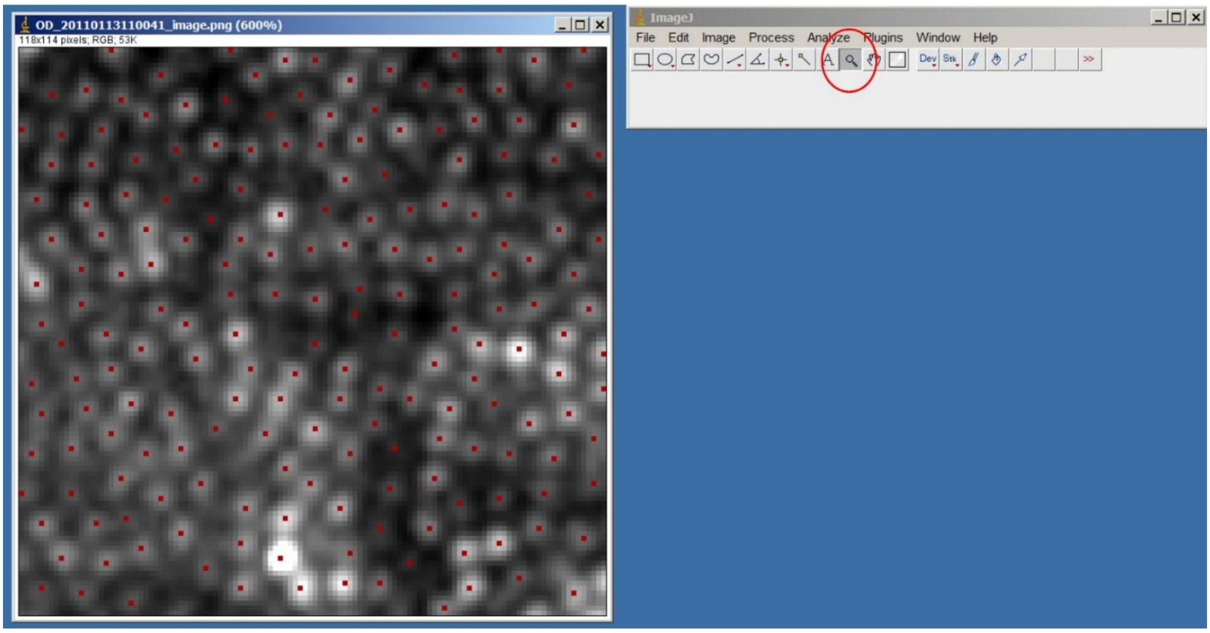

2) Open the cell counter plugin

Scroll down the Plugins menu to select Cell Counter (sub-fovea, 1.5 and 3 mm nasal and temporal to the fovea) and placed it such that the central point on the grid coincides with the reference line.

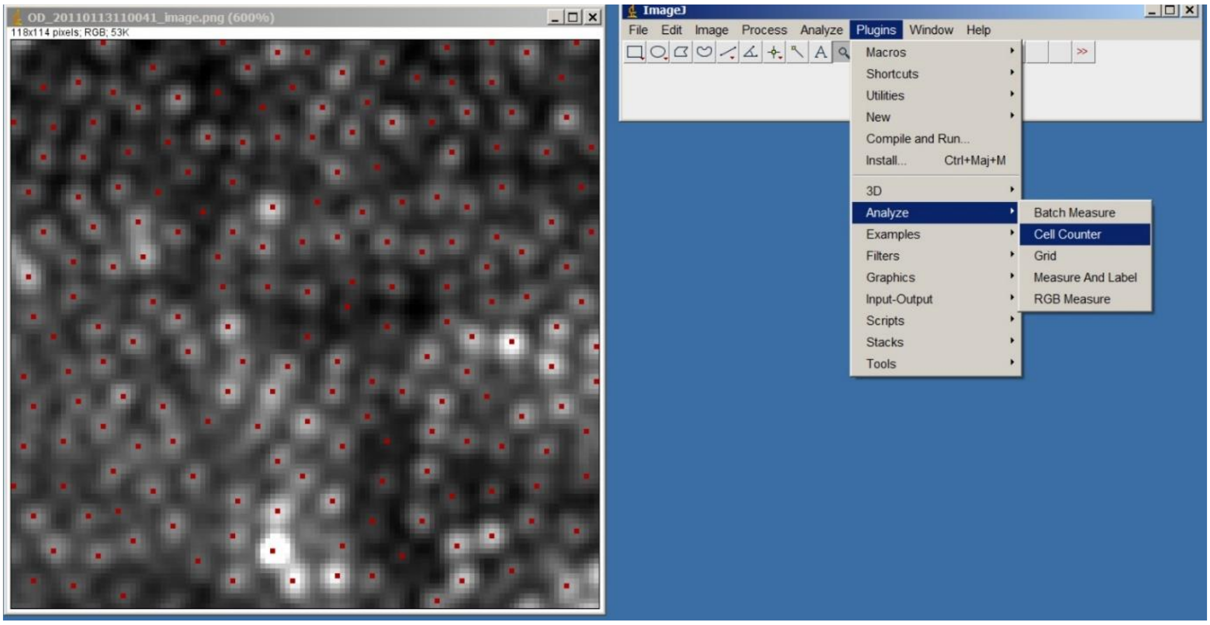

In the following window, select initialize

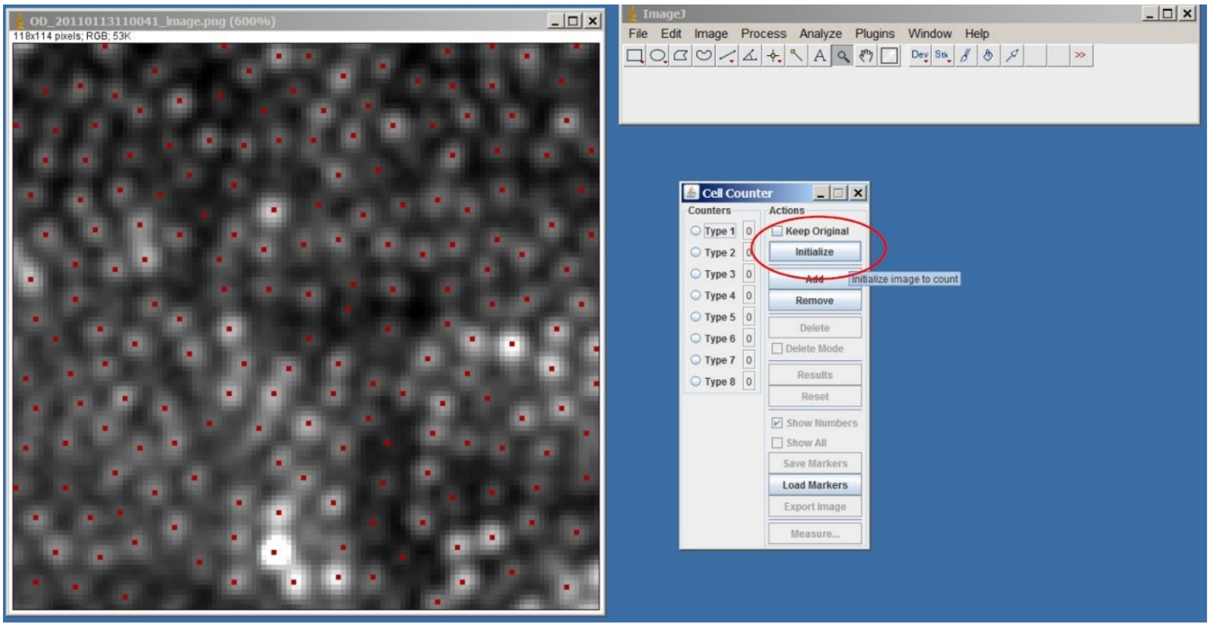

3). Start counting

Select the paintbrush tool (red circle), then click on Type 1 (red oval). You can start selecting the cells you want to remove (yellow circles). The number of "deleted" cells will increment as you select them (in the counter box next to Type 1).

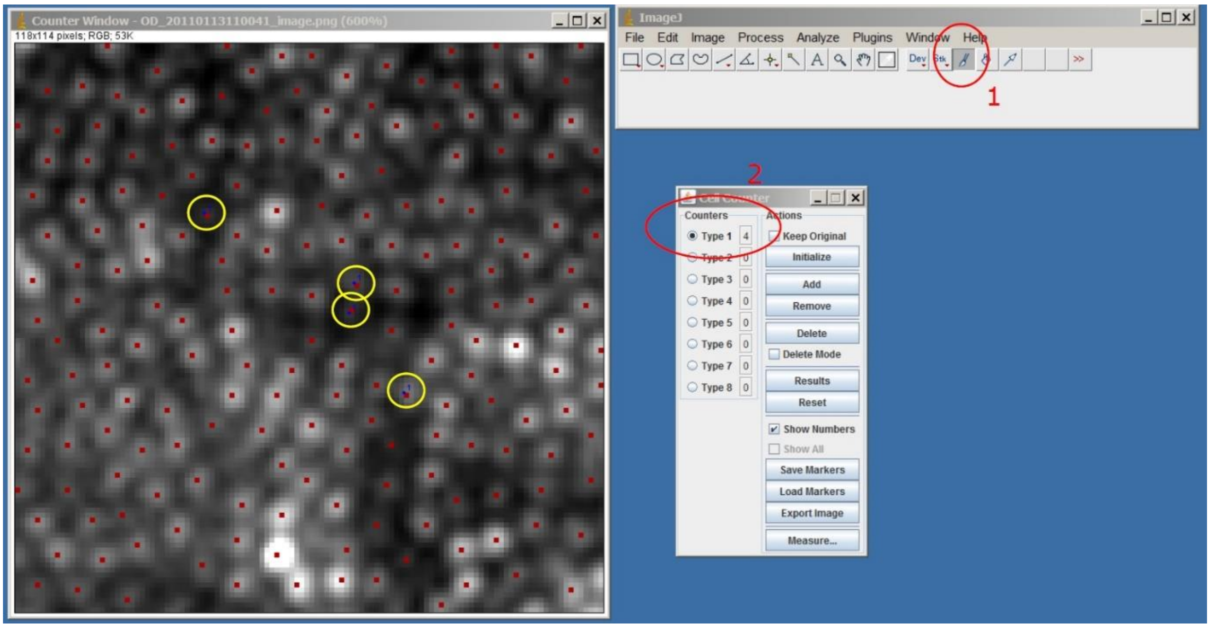

Once this selection done, click on Type 2 and start selecting the cells that you want to add.

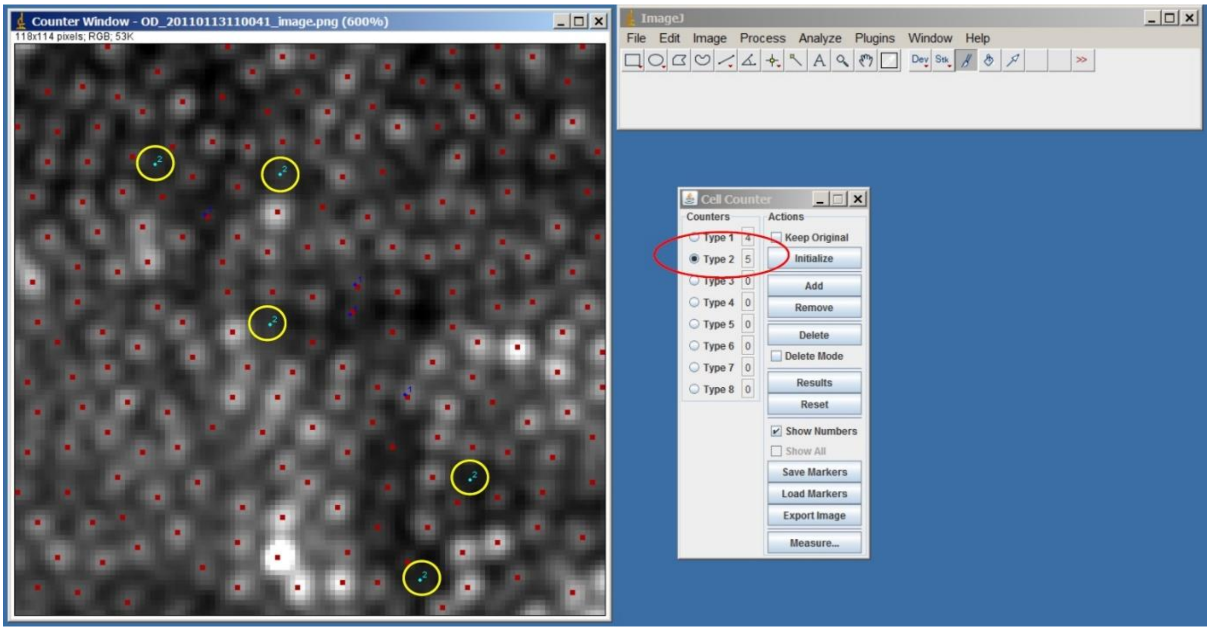

Supplement: S1 Appendix — (PDF) [file pone.0152788.s001.pdf]
